# Sichuan pepper, *Zanthoxylum bungeanum* Maxim., suppresses human coronavirus OC43 infection by inhibiting viral entry and impairing autolysosome accumulation

**DOI:** 10.1186/s13020-026-01388-y

**Published:** 2026-04-02

**Authors:** Hyeonjeong Park, In Jin Ha, Young-Hee Jin, Sunoh Kwon

**Affiliations:** 1 Department of Science in Korean Medicine, Graduate School, Kyung Hee University, Seoul, 02447 Republic of Korea; 2 Korean Medicine Convergence Research Division, Korea Institute of Oriental Medicine, Daejeon, 34054 Republic of Korea; 3 Korean Medicine Clinical Trial Center (K-CTC), Kyung Hee University Korean Medicine Hospital, Seoul, 02454 Republic of Korea; 4 Korean Medicine Application Center, Korea Institute of Oriental Medicine, Daegu, 41062 Republic of Korea; 5 KIOM School, University of Science and Technology (UST), Daejeon, 34113 Republic of Korea

**Keywords:** Coronavirus, HCoV-OC43, Autophagy, Autolysosome, Antiviral agents, *Zanthoxylum bungeanum*

## Abstract

**Background:**

The dried pericarp of *Zanthoxylum bungeanum* Maxim. (Rutaceae), widely recognized as Sichuan pepper (*Hua Jiao*), is a prominent herb within the Traditional Chinese Medicine (TCM), boasting a long and well-documented history of ethnopharmacological application. Its traditional uses primarily involve the internal management of gastrointestinal ailments, such as stomachaches, alongside promoting systemic blood circulation. Building on this traditional foundation, modern pharmacological studies have confirmed and extended its therapeutic profile, revealing potent anti-inflammatory and antitumor biological activities. We examined the anticoronaviral effects and the mechanism of action of the 30% ethanol extract of *Z. bungeanum* against human coronavirus (HCoV)-OC43 infection.

**Methods:**

Antiviral activity was measured using the virus-induced cytopathic effect (CPE) reduction assay, qRT-PCR was performed to calculate viral RNA copy numbers, and western blotting and immunofluorescence staining were performed to detect viral proteins. To evaluate the mode of action of *Z. bungeanum*, time-of-addition, attachment, penetration, and virucidal assays were performed. The effect of the extract on virus-induced autophagic flux was examined by measuring LC3 protein expression via western blotting, and autophagy, lysosomes, and lysosomal degradation were assessed using CYTO-ID® Green, LysoTracker™ Deep Red, and DQ™ Red BSA, respectively. Ultra-performance liquid chromatography quadrupole time-of-flight mass spectrometry was used to identify the active components in the extract.

**Results:**

*Z. bungeanum* extract protected against HCoV-OC43-induced CPEs with IC_50_ of 284.1 ± 44.0 µg/mL. The extract reduced the number of intracellular and extracellular viral RNA copy and viral protein expression. *Z. bungeanum* mainly affected the early phase of the virus life cycle by inhibiting viral entry. Additionally, *Z. bungeanum* inhibited HCoV-OC43 replication by inducing autolysosome accumulation, thereby blocking virus-induced autophagic flux. Hydroxy-α-sanshool and *p*-coumaric acid were identified as the active antiviral components.

**Conclusions:**

This study suggested the potential of *Z. bungeanum* as a novel anticoronaviral agent.

**Graphical abstract:**

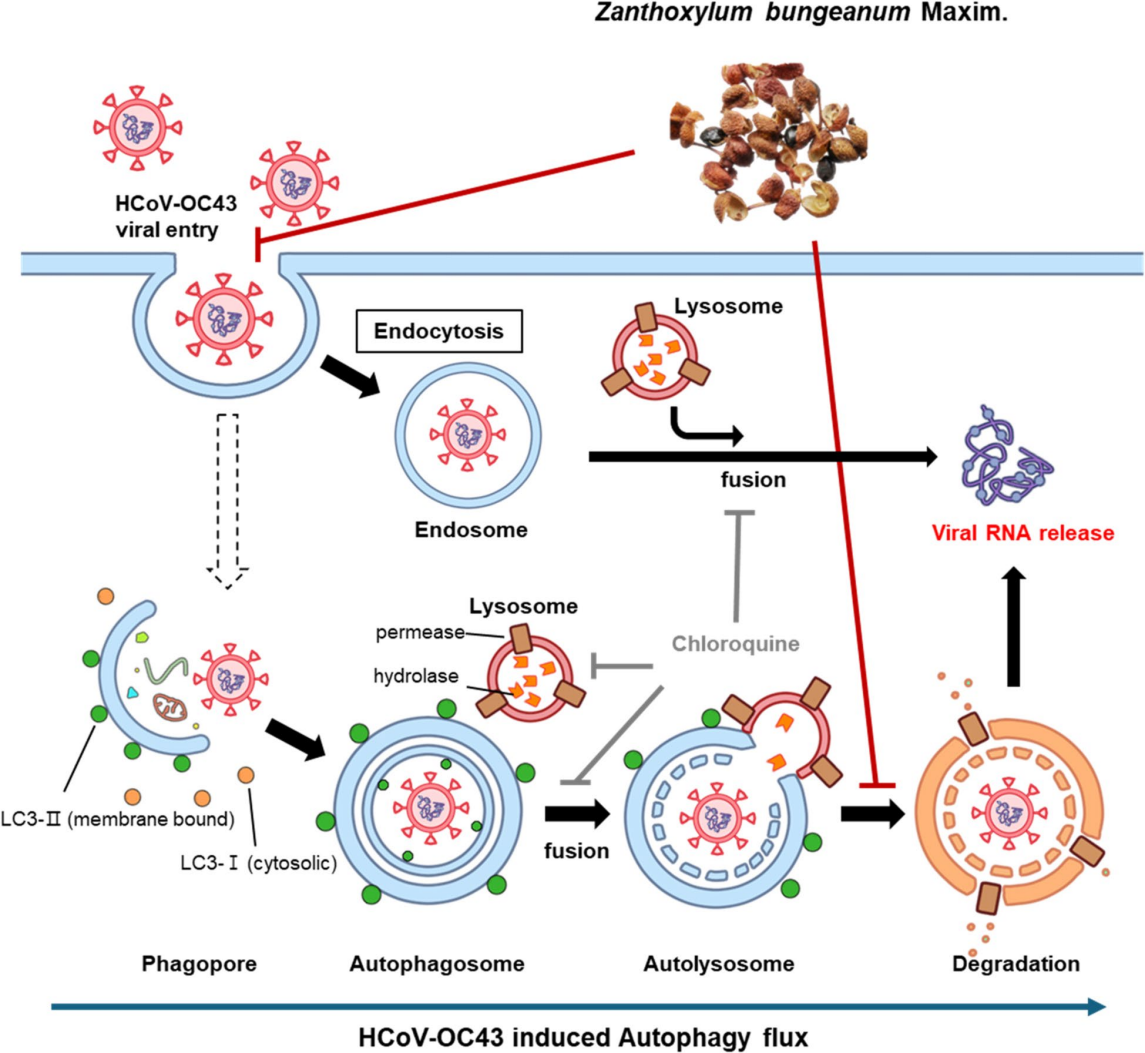

**Supplementary Information:**

The online version contains supplementary material available at 10.1186/s13020-026-01388-y.

## Highlights

- Chinese pepper, *Zanthoxylum bungeanum* Maxim., inhibits HCoV-OC43 infection.
- *Z. bungeanum* blocks the viral entry.
- *Z. bungeanum* inhibits viral infection by the accumulation of impaired autolysosomes.
- Hydroxy-α-sanshool and *p*-coumaric acid were the active compounds in *Z. bungeanum*.
- *Z. bungeanum* emerged as a therapeutic agent for coronavirus infection.

**Supplementary Information:**

The online version contains supplementary material available at 10.1186/s13020-026-01388-y.

## Introduction

Seven human coronaviruses (HCoVs) have been discovered thus far, including HCoV-NL63 and HCoV-229E in *Alphacoronavirus* and HCoV-HKU1, HCoV-OC43, SARS-CoV, MERS-CoV, and SARS-CoV-2 in *Betacoronavirus*, which are among the four genera, *Alpha-, Beta-, Gamma- and Delta-coronavirus* of the enveloped, positive-sense single-stranded RNA coronaviruses in the family *Coronaviridae* [1]. HCoV-OC43, isolated in 1967 from the tracheal secretions of a patient, is the most frequently detected HCoV [2]. It replicates in epithelial cells in the upper respiratory tract and commonly triggers mild cold symptoms such as cough and sore throat [3]. SARS-CoV-2 emerged in 2019 and caused the COVID-19 pandemic, resulting in an estimated 7.1 million cumulative deaths globally [4]. Although Veklury, Paxlovid, and Lagevrio were developed as COVID-19 agents targeting viral proteins, RNA-dependent RNA polymerase, and main protease, antiviral–resistant coronavirus variants have continuously emerged [5, 6]. This emphasizes the importance of developing antiviral agents with alternative mechanisms of action to combat the newly emerging coronavirus.

Therapeutics targeting the host machinery viruses utilize for replication exhibit broad-spectrum antiviral activity and carry a lower risk of drug resistance [7]. Autophagy is a conserved cellular process of lysosomal degradation that maintains homeostasis, and it plays a dual role in the response to viral infection [8]. Specifically, autophagy contributes to viral clearance by degrading viral components and particles and promotes an innate immune response by stimulating interactions between pattern recognition receptors and various autophagic components [9, 10]. RNA viruses can utilize autophagy to their own benefit by forming a double-membrane compartment as a replication platform to evade the host immune system [8]. Coxsackievirus B3, hepatitis C virus, influenza A virus, and foot-and-mouth disease virus have been reported to facilitate autophagy to promote viral replication [11–14]. Coronaviruses, including SARS-CoV and MERS-CoV, induce the development of double-membrane vesicles as viral replication organelles [15, 16]. HCoV-OC43 has been reported to induce autophagic flux, and the impairment of virus-induced autophagy significantly reduced viral infection [17]. Specifically, hydroxychloroquine and chloroquine as autophagy inhibitors impede SARS-CoV-2 replication [18, 19]. As viruses exploit autophagy processes, autophagy could represent a target for therapeutic intervention.

*Zanthoxylum bungeanum* Maxim*.* belongs to the Rutaceae family, and it is widely used as a spice and as a traditional medicine for pain, diarrhea, coldness, and dampness [20]. The anti-inflammatory activity of *Z. bungeanum* is exerted through the TLR4 signaling pathways [21]. *Z. bungeanum* exhibits antitumor effects by regulating various host signaling pathways, such as the CDC25A/cyclin B1/CDK1 and p53 pathways [22, 23]. *Z. bungeanum* modulates autophagy via PI3K/AKT/mTOR signaling and protects against UVB-induced photodamage [24, 25]. However, the antiviral activity of *Z. bungeanum* through autophagy regulation and its therapeutic potential remain unknown.

In this study, we described the significant antiviral effect of *Z. bungeanum* on HCoV-OC43 infection through its ability to inhibit viral entry and disrupt virus-induced autophagy flux, especially impaired autolysosome accumulation. We identified hydroxy-α-sanshool and *p-*coumaric acid as the active antiviral compounds of *Z. bungeanum*. Our findings suggest the therapeutic potential of *Z. bungeanum* as a novel anticoronavirus agent through its modulation of virus-induced autophagy.

## Materials and methods

### Extract and compounds

A 30% ethanol extract of *Z. bungeanum* was prepared via heat reflux extraction of 500 g of the dried pericarp of *Z. bungeanum* Maxim. (Omniherb Co., Ltd., Uiseong, Korea) and 5 L of 30% ethanol at 94 °C for 3 h. The extract was filtered, concentrated under reduced pressure at 50 °C, and subsequently lyophilized (extract yield, 14.83%). Hydroxy-α-sanshool (PubChem CID 10084135; Wuhan ChemFaces Biochemical Co., Ltd., Wuhan, China) and *p*-coumaric acid (PubChem CID 24893127; Chengdu Must Bio-Technology Co., Ltd., Chengdu, China) were solubilized to create 100-mM stock solutions. Chloroquine and rapamycin were purchased from Sigma-Aldrich.

### Qualification and quantification of chemical components by UHPLC-QTOF MS analysis

**Fig. 1:**
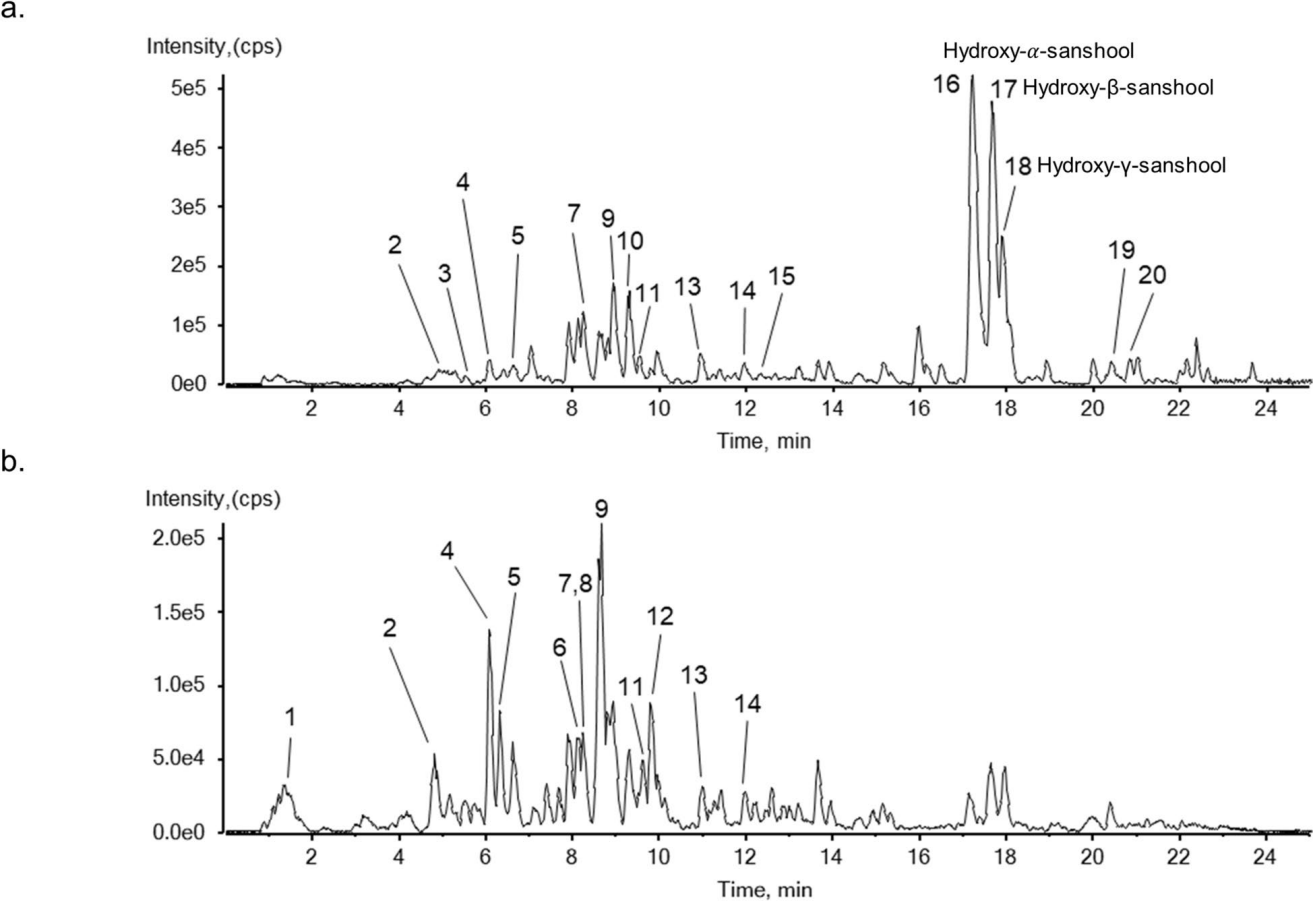
Representative base peak chromatogram of the 30% ethanol extract of *Z. bungeanum* positive ionization mode (**a**) and negative ionization mode (**b**) using UHPLC-ESI-QTOF MS/MS analysis

A chromatographic analysis of *Z. bungeanum* was performed using UHPLC-QTOF MS to qualify the chemical components, and twenty compounds were identified in the 30% ethanol extract of *Z. bungeanum* (Fig. 1 and Table 1). UHPLC (Thermo Fisher Scientific, Sunnyvale, CA, USA) with an ACQUITY UPLC HSS T3 column (2.1 × 100 mm, 1.8 µm) was coupled to a Triple TOF5600 + (SCIEX, Foster City, CA, USA) with an electrospray ionization (ESI) source operated in the positive/negative ion modes as described in supplementary methods.

**Table 1:** Identification of chemical components in the extract based on UHPLC-QTOF MS/MS

No	Name	Formula	mass (Da)	Expected	Adduct	Found at	Error	MS/MS product ions	Identified with
				RT (min)		mass (Da)	(ppm)		
1	Arbutin	C_12_H_16_O_7_	272.0896	1.65	[M-H] ^−^	271.0823	−0.1	108.0209, 109.0295, 96.9594	^#^
2	Neochlorogenic acid	C_16_H_18_O_9_	354.0951	4.80	[M + H] ^+^	355.1025	0.5	163.0391, 145.0282, 135.0440	^#^
					[M-H] ^−^	353.0875	−0.8	191.0562, 179.0350, 135.0453	
3	Coumarin	C_9_H_6_O_2_	146.0368	5.80	[M + H] ^+^	147.0438	−1.5	91.0534, 65.0375, 119.0567	^#^
4	Cryptochlorogenic acid	C_16_H_18_O_9_	354.0951	6.07	[M + H] ^+^	355.1026	0.7	163.0390, 145.0284, 117.0339	^#^
					[M-H] ^−^	353.0872	−1.7	191.0562, 173.0457, 85.0307	
5	Chlorogenic acid	C_16_H_18_O_9_	354.0951	6.31	[M + H] ^+^	355.1023	−0.1	163.0384, 145.0278, 117.0328	^#^
					[M-H] ^−^	353.0877	−0.4	191.0560, 173.0454, 179.0347, 135.0454	
6	*p*-Coumaric acid	C_9_H_8_O_3_	164.0473	7.97	[M-H] ^−^	163.0402	0.9	119.0505, 93.0346, 117.0361, 89.0395	^†^
7	Umbelliferone	C_9_H_6_O_3_	162.0317	8.42	[M + H] ^+^	163.0391	0.6	No MS/MS	^#^
					[M-H] ^−^	161.0246	1	133.0296, 105.0345, 77.0429	
8	3-Hydroxycinnamic acid	C_9_H_8_O_3_	164.0473	8.42	[M-H] ^−^	163.0399	−0.9	119.0491, 93.034, 117.0331, 91.0563	^#^
9	Hyperoside	C_21_H_20_O_12_	464.0955	8.81	[M + H] ^+^	465.1025	−0.6	303.0503	^#^
					[M-H] ^−^	463.0878	−0.9	300.0273, 301.0351, 271.0246	
10	( ±)-ZP-amide N	C_16_H_27_NO_4_	297.1940	9.28	[M + H] ^+^	298.2015	0.8	105.0704, 149.0959, 262.1804	^#^
11	3,5-dicaffeoylquinic acid	C_25_H_24_O_12_	516.1268	9.67	[M + H] ^+^	517.1338	−0.5	163.0387, 145.0286, 135.0433, 499.1206	^#^
					[M-H] ^−^	515.1192	−0.6	353.0871, 191.0550, 179.0341	
12	4,5-dicaffeoylquinic acid	C_25_H_24_O_12_	516.1268	10.17	[M + H] ^+^	517.1343	0.5	163.0386, 145.0287, 135.0439, 499.1235	^#^
					[M-H] ^−^	515.1199	0.8	353.0872, 173.0455, 179.0336, 191.0544	
13	Isorhamnetin 7-O-glucoside	C_22_H_22_O_12_	478.1111	11.06	[M + H] ^+^	479.1179	1.0	317.0669	^#^
					[M-H] ^−^	477.1035	−0.8	314.0432, 271.0248	
14	Quercetin	C_15_H_10_O_7_	302.0427	12.19	[M + H] ^+^	303.0501	0.7	153.0183, 229.0498, 257.0445, 137.0236	^#^
					[M-H] ^−^	301.0355	−0.1	151.0036, 121.0302, 107.0144	
15	7-methoxy coumarin	C_10_H_8_O_3_	176.0473	12.35	[M + H] ^+^	177.0545	0.8	121.0651, 177.0538, 133.0634, 93.0349	^#^
16	Hydroxy-α-sanshool	C_16_H_25_O_2_	264.1958	17.66	[M + H] ^+^	264.1955	−1.1	79.0558, 91.0551, 107.0856, 246.7847	^†^
17	Hydroxy-β-sanshool	C_16_H_25_O_2_	264.1958	17.39	[M + H] ^+^	264.1956	−0.8	79.0565, 91.0558, 107.0863, 246.1856	^#^
18	Hydroxy-γ-sanshool	C_16_H_25_O_2_	264.1958	17.90	[M + H] ^+^	264.1956	−1.0	No MS/MS	^#^
19	(2E,4E,8Z,11E)−2′-hydroxy-N-isobutyl-2,4,8,11-tetradecatetraenamide	C_18_H_29_NO_2_	291.2198	20.49	[M + H] ^+^	292.2271	0.0	274.2169, 131.0850, 91.0563, 67.0568	^*^
20	α-sanshool	C_16_H_25_NO	247.1936	20.83	[M + H] ^+^	248.2007	−0.8	79.0564, 91.0551, 107.0861	^*^

^#^ In-house ms/ms library and online database; such as GNPS, MASS bank or Metlin

^†^ Reference standard

^*^ Extract MS with isotope mass

It has been reported that hydroxy-α-sanshool, as the main alkylamides [26, 27] and *p*-coumaric acid [28], are present in the extract of *Z. bungeanum.* We developed and validated the reliable UHPLC-QTOF MS/MS method with MRM^HR^ (multiple reaction monitoring) scan for simultaneous quantification of active antiviral compounds, hydroxy-α-sanshool, and *p*-coumaric acid in the 30% ethanol extract of *Z. bungeanum* in accordance with European Medicines Agency (EMA) guidelines and the relevant guidelines as described in supplementary methods and supplementary results (Additional file 1: Table S1, S2, and S3). The concentrations of hydroxy-α-sanshool and *p*-coumaric acid were calculated as 144.13 ± 2.15 mg/g and 0.09 ± 0.00 mg/g, respectively (Table 2).

**Table 2:** Quantification results of hydroxy-α-sanshool and *p*-coumaric acid in this analysis

No	Name	Concentration (mg/g)^a^	% CV
1	Hydroxy-α-sanshool	144.13 ± 2.15	1.50
2	*p*-Coumaric acid	0.09 ± 0.00	0.27

^a^ Mean ± S.D, n = 3 (batch)

### Cells and virus

MRC-5 cells (ATCC, Manassas, VA, USA) were grown in MEM (Corning Inc., Corning, NY, USA) containing 10% FBS (Cytiva, Marlborough, MA, USA) and antibiotic solution. (Gibco, Carlsbad, CA, USA) at 37 °C with 5% CO_2_. HCoV-OC43 (ATCC) was propagated and titrated as previously described [29]. MRC-5 cells were inoculated with 0.1 multiplicity of infection (MOI) HCoV-OC43 (1 × 10^4.5^ TCID_50_/mL) in medium containing 2% FBS at 33 °C in a humidified atmosphere containing 5% CO_2_.

### Cytotoxicity assay

Cell viability was quantified using an MTS-based assay (Promega Corporation, Madison, WI, USA). Absorbance was detected at 490 nm using a GM3000 GloMax® Discover Microplate Reader (Promega Corporation). To assess viability, the absorbance values were normalized using the 10% DMSO-treated control (0% viability) and vehicle-treated control (100% viability).

### Cytopathic effect (CPE) reduction assay

After HCoV-OC43–infected MRC-5 cells were treated with *Z. bungeanum*, the MTS assay was conducted as previously described. Absorbance values were normalized using the infected vehicle control (0%) and uninfected vehicle control (100%).

### Quantitative RT-PCR (qRT-PCR)

The RNeasy® kit (Qiagen N.V., Hilden, Germany) was used to isolate total RNA from the cell lysate. Viral RNA from culture supernatants was extracted with the QIAamp Viral RNA Kit (Qiagen). qRT-PCR was conducted using the One-Step RT-PCR Kit (Takara Bio Inc., Kusatsu, Japan) and the CFX Opus 96 Real-time PCR (Bio-Rad Laboratories, Inc, Hercules, CA, USA). The sequences of the primers targeting the HCoV-OC43 nucleocapsid protein and the standard curve (tenfold of nucleocapsid protein RNA as 10^10^ to 10^1^ copies/µL) of the viral RNA copy number were used for the calculation of the sample’s viral RNA copy numbers as previously described [29].

### Western blotting

MRC-5 cells were lysed in RIPA lysis buffer. After SDS-PAGE and dry transfer onto a nitrocellulose membrane (Bio-Rad Laboratories), the membranes blocking with EveryBlot Blocking buffer (Bio-Rad) were reacted with primary antibodies against HCoV-OC43 spike protein (CusaBio Technology LLC, Houston, TX, USA), HCoV-OC43 nucleocapsid protein (Merck & Co., Inc., Rahway, NJ, USA), LC3B (Abcam PLC, Cambridge, UK), SQSTM1/p62 (Abcam), beclin-1 (BioLegend, San Diego, CA, USA), and β-actin (Cell Signaling Technology, Danvers, MA, USA), followed by secondary HRP-linked antibodies (Abcam). Protein bands were visualized using the ChemiDoc™ MP Imaging System (Bio-Rad). Band intensity was analyzed using Image Lab 6.1 (Bio-Rad), and each value was normalized against a loading control.

### Immunofluorescence staining of viral proteins

MRC-5 cells were fixed and permeabilized with 4% paraformaldehyde solution and 0.2% Triton X-100, respectively. After blocking with 3% BSA and 0.2% Triton X-100 in PBS, cells were reacted with anti-HCoV-OC43 nucleocapsid protein (Merck & Co.) or anti- HCoV-OC43 spike protein antibody (Cusabio), followed by secondary antibody (Thermo Fisher Scientific). Fluorescence images were captured using a CKX53 fluorescence microscope (Olympus Corporation, Tokyo, Japan).

### Viral plaque assay

MRC-5 cells in 24-well plates were inoculated with 100 μL/well of a tenfold serial dilution of HCoV-OC43 in MEM supplemented with 2% FBS with or without *Z. bungeanum* extract for 2 h at 33 °C. The inoculum was removed, and the cells were overlaid with 0.75% agarose (Lonza, Basel, Switzerland) in 2% FBS MEM for 4 days post-infection at 33 °C. Cells were fixed with 4% paraformaldehyde solution and stained with 0.5% crystal violet solution.

### Time-of-addition assay

In the pretreatment assay, MRC-5 cells were pretreated with the *Z. bungeanum* extract (100–1000 μg/mL) for 4 h. After a washing step to remove the extract, the cells were infected with HCoV-OC43. For the co-treatment assay, cells were simultaneously infected with the virus and treated with *Z. bungeanum* extract for 4 h. In the post-treatment assay, the cells were infected with the virus for 4 h and treated with *Z. bungeanum* extract. After all assays, cell viability was analyzed using the MTS assay at 4 days post-infection (dpi).

### Viral attachment, penetration, and virucidal assays

The viral attachment assay was conducted using MRC-5 cells after virus infection and *Z. bungeanum* extract treatment for 1 h at 4 °C to enable attachment while inhibiting viral internalization, after which unbound virus and extract were removed via washing. Treated cells were then incubated at 33 °C for 24 h to permit viral entry. For the penetration assay, the virus was allowed to attach to the cells at 4 °C for 1 h, after which unbound viral particles were removed. Cells were treated with the extract at 33 °C for 1 h, after which the residual extract was removed. To perform the virucidal assay, the virus was mixed with the extract and incubated for 4 h at 33 °C. Subsequently, the cells were inoculated with the virus–extract mixture at 33 °C for 1 h, followed by a washing step to remove the mixture. Total RNA was extracted at 1 dpi, and viral RNA copies were counted using qRT-PCR.

### Fluorescence analysis of autophagy, lysosomes, and lysosomal degradation

To visualize autophagy and lysosomes, cells were treated with 50 nM LysoTracker™ Deep Red (Invitrogen Corporation, Waltham, MA, USA) for 30 min and washed. Subsequently, cells were treated with CYTO-ID® Green detection reagent (Enzo Life Sciences, Farmingdale, NY, USA) and Hoechst 33342 (Invitrogen) for 30 min at 37 °C. To measure lysosomal degradation, cells were incubated with DQ™ Red BSA fluorescence substrate (Invitrogen) for 3 h at 37 °C and stained with CYTO-ID Green detection reagent for 30 min at 37 °C. Cells were fixed with 4% paraformaldehyde solution for 20 min and mounted on a glass slide using SlowFade Gold antifade reagent with DAPI (Invitrogen). Fluorescence images were taken using an LSM 700 confocal laser-scanning microscope (Carl Zeiss AG, Oberkochen, Germany) and ZEN software (Carl Zeiss). Puncta were counted and fluorescence mean intensity was measured using Fiji/ImageJ Software (US National Institutes of Health, Bethesda, MD, USA).

### Statistical analysis

Data are presented as the mean ± SD, and statistical analysis was conducted using GraphPad Prism 10.4.2 Software (GraphPad Software, Inc., San Diego, CA, USA). Statistical significance was assessed using Student’s *t*-test or one-way ANOVA with Dunnett’s multiple comparisons test.

## Results

### Z. bungeanum exhibits potent antiviral activity against HCoV-OC43

**Fig. 2:**
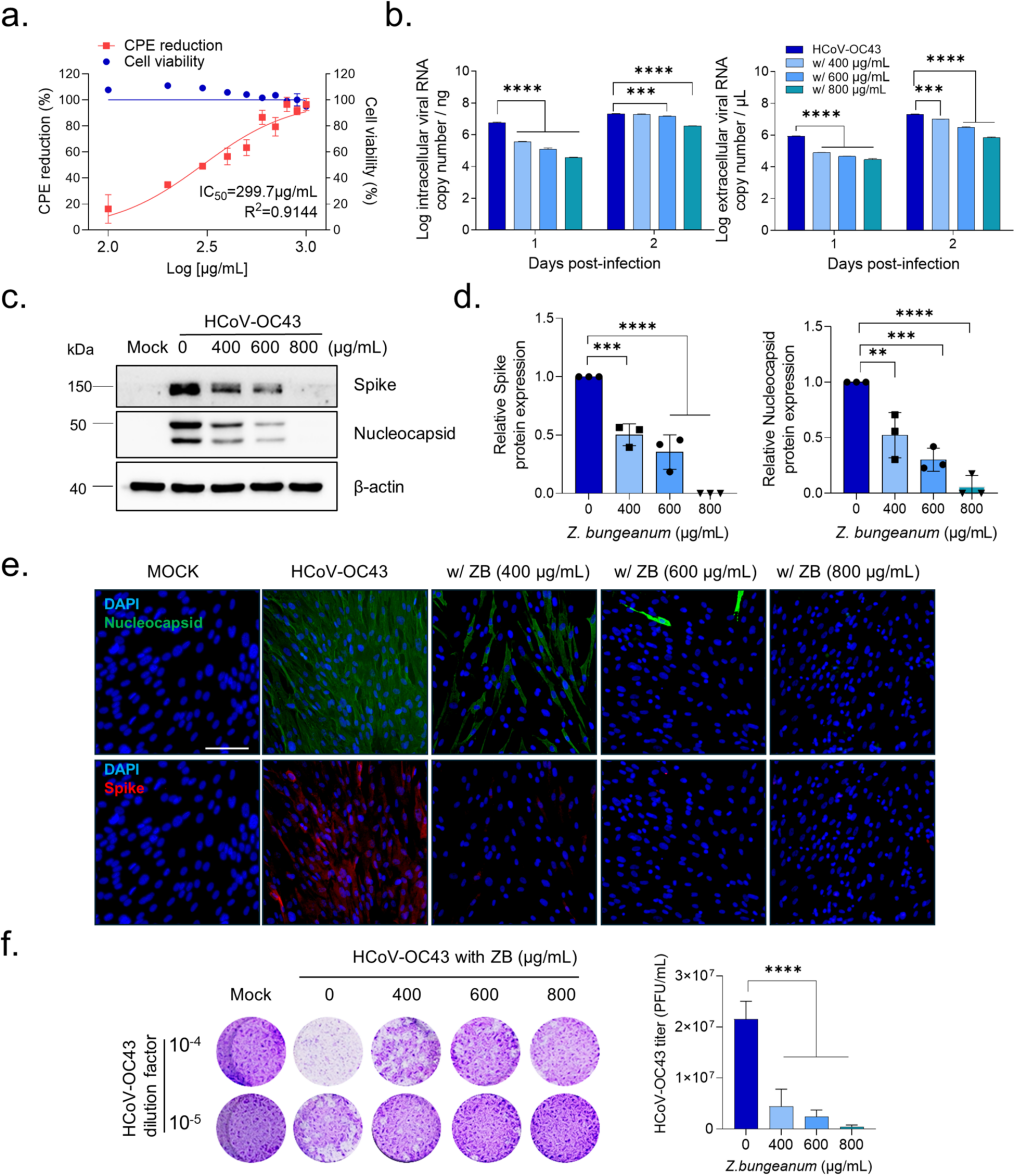
*Z. bungeanum* inhibits HCoV-OC43 infection. **a** MRC-5 cells were treated with *Z. bungeanum* for 24 h with or without HCoV-OC43. Cell viability and CPE rates were determined using the MTS assay at 4 dpi. **b** Viral RNA copy number in the cell lysate (left panel) and supernatant (right panel) of virus-infected cells treated with *Z. bungeanum* was measured using RT-qPCR. **c** HCoV-OC43 spike protein and nucleocapsid protein expression was determined in virus-infected cells treated with *Z. bungeanum* by western blotting at 2 dpi. **d** Relative expression of the viral spike (left panel) and nucleocapsid proteins (right panel) was normalized to β-actin expression. **e** Immunofluorescence images of HCoV-OC43–infected MRC-5 cells treated with *Z. bungeanum* (ZB) at 2 dpi. DAPI, blue; nucleocapsid protein, green; spike protein, red. Scale bar: 100 μm. **f** MRC-5 cells were inoculated with serial dilutions of the HCoV-OC43 virus (10^–4^ to 10^–5^) with or without ZB (400, 600, and 800 µg/mL). Viral plaques were stained, and viral titers, plaque forming unit (PFU)/mL were determined at 4 dpi. One-way ANOVA with Dunnett’s multiple comparisons was used to assess statistical significance: *p < 0.05, **p < 0.01, ***p < 0.001, ****p < 0.0001 versus control. Data represent at least three independent experiments

MRC-5 cells were uninfected or infected with HCoV-OC43 with *Z. bungeanum* (100 μg/mL–1000 μg/mL) for 24 h, and cell viability was evaluated using MTS assay at 4 dpi (Fig. 2a). *Z. bungeanum* extract did not induce cytotoxicity at concentrations lower than 800 μg/mL and completely protected from the virus-induced cell death above 800 μg/mL (Fig. 2a). The extract protected against HCoV-OC43–induced CPEs with a 50% inhibitory concentration of 284.1 ± 44.0 μg/mL. *Z. bungeanum* extract concentration-dependently reduced viral RNA copy numbers in the cell lysate and cell culture supernatant (Fig. 2b), indicating the inhibition of intracellular viral replication and extracellular virus release. *Z. bungeanum* reduced the expression of HCoV-OC43 spike protein and nucleocapsid protein at 2 dpi (Fig. 2c, 2d). Immunofluorescence staining further illustrated that *Z. bungeanum* inhibited spike protein and nucleocapsid protein expression (Fig. 2e). We also confirmed that *Z. bungeanum* markedly inhibited the HCoV-OC43-induced plaque formation in the viral plaque reduction assay, suggesting that *Z. bungeanum* reduced infectious virion production (Fig. 2f). These results suggest that *Z. bungeanum* has anti-HCoV-OC43 activity.

### Z. bungeanum inhibits HCoV-OC43 infection during the initial phase of the virus life cycle by interfering with viral entry

**Fig. 3:**
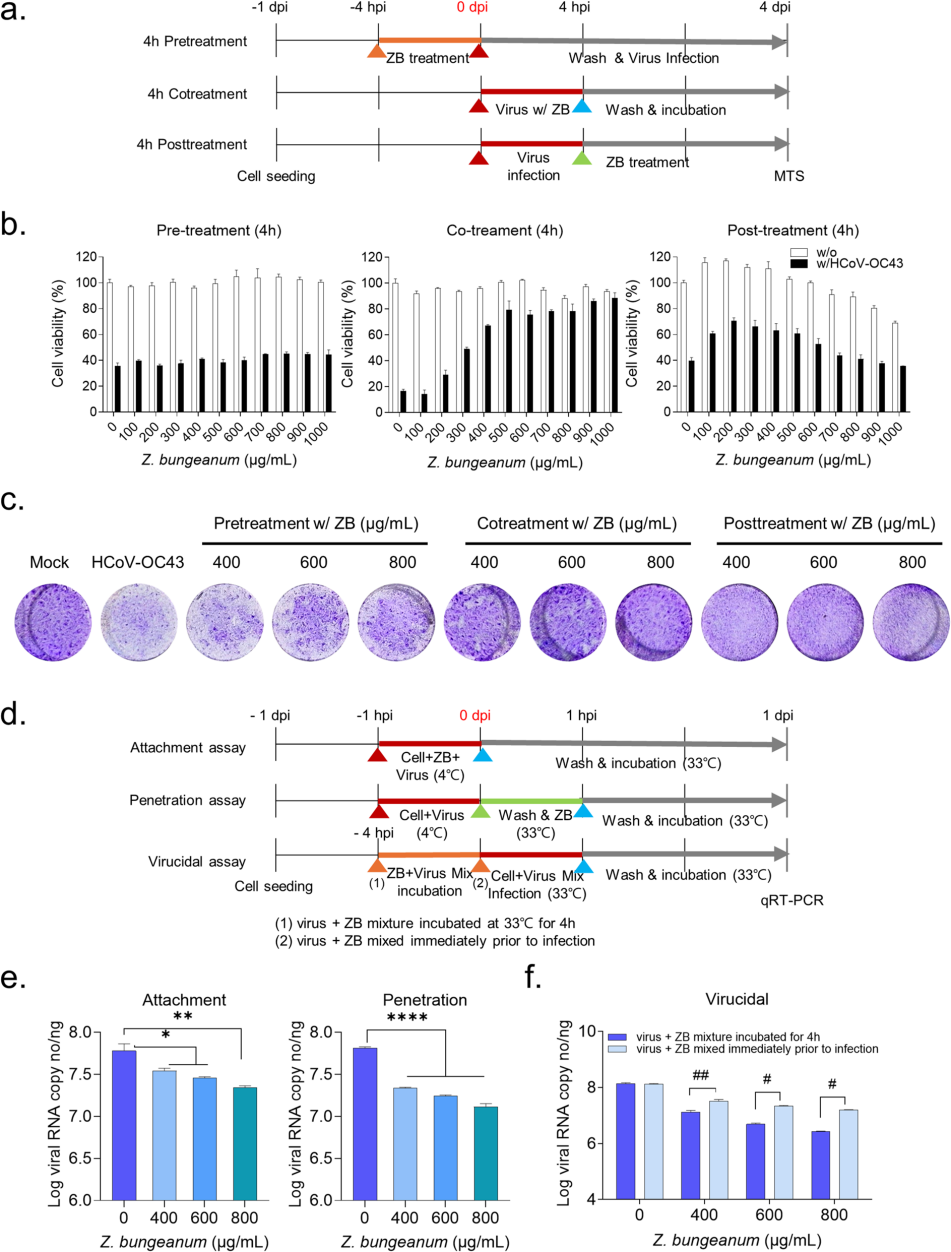
*Z. bungeanum* inhibits the viral entry in the early phase of the viral life cycle. **a** Overview of the pretreatment, co-treatment, and post-treatment of *Z. bungeanum* (ZB) experimental design. **b** MRC-5 cells were treated with *Z. bungeanum* at 4 h prior to infection (left panel), co-treated with *Z. bungeanum* and the virus for 4 h (middle panel), or treated with *Z. bungeanum* at 4 h after infection (right panel). Cell viability was detected using the MTS assay at 4 dpi. **c** Viral plaque reduction effect of *Z. bungeanum* in the time-of-addition assay. MRC-5 cells were pre-treated for 2 h, co-treated with *Z. bungeanum* and the virus for 2 h, or post-treated with *Z. bungeanum* at 2 h after infection (10^–4^ dilution). Viral plaques were stained at 4 dpi. **d** Overview of the HCoV-OC43 attachment, penetration, and virucidal assays. **e** and **f** Viral RNA copy numbers were determined in the cell lysate from the attachment, penetration (**d**), and virucidal (**e**) assays at 1 dpi using RT-qPCR. Statistical analysis was performed using one-way ANOVA with Dunnett’s multiple comparisons (*p < 0.05, **p < 0.01, ****p < 0.0001) and Student’s *t*-test (#*p* < 0.05, ##*p* < 0.01). Data represent three independent experiments

To examine which phase of the viral life cycle *Z. bungeanum* targets, a time-of-addition assay (100 µg/mL–1000 µg/mL) was performed (Fig. 3a). Pretreatment with *Z. bungeanum* for 4 h failed to prevent CPEs and *Z. bungeanum* co-treatment for 4 h concentration-dependently increased cell viability, indicating a reduction in CPEs. Post-treatment with *Z. bungeanum* for 4 h slightly prevented virus-induced cell death, but these effects were attenuated by the cytotoxicity of the extract (Fig. 3b). The plaque reduction assay also showed the marked inhibition of infectious virion in co-treatment with *Z. bungeanum,* although a modest reduction in viral plaques was detected in the pre-treatment assay and the cytotoxicity was shown in the post-treatment assay (Fig. 3c). These results demonstrate that *Z. bungeanum* exhibits effective antiviral activity during the initial phase of viral infection*.*

To clarify the early phase of viral infection, the ability of *Z. bungeanum* to inhibit virus attachment and penetration and its virucidal activity were measured (Fig. 3d). *Z. bungeanum* significantly reduced viral RNA copy numbers in the attachment and penetration assay (Fig. 3e), indicating its ability to inhibit viral attachment and internalization into host cells. In the virucidal assay, exposure of MRC-5 cells to the virus–extract mixture that was incubated for 4 h significantly reduced viral RNA copy numbers in comparison to the effect of the virus–extract mixture for which the incubation step was omitted, suggesting that *Z. bungeanum* has a direct virus particle sterilization effect in addition to its inhibition of viral entry (Fig. 3f). These results suggest that *Z. bungeanum* interferes with virus attachment and penetration into host cells and directly disrupts virus particles.

### Z. bungeanum impedes HCoV-OC43–promoted autophagic flux in MRC-5 cells

**Fig. 4:**
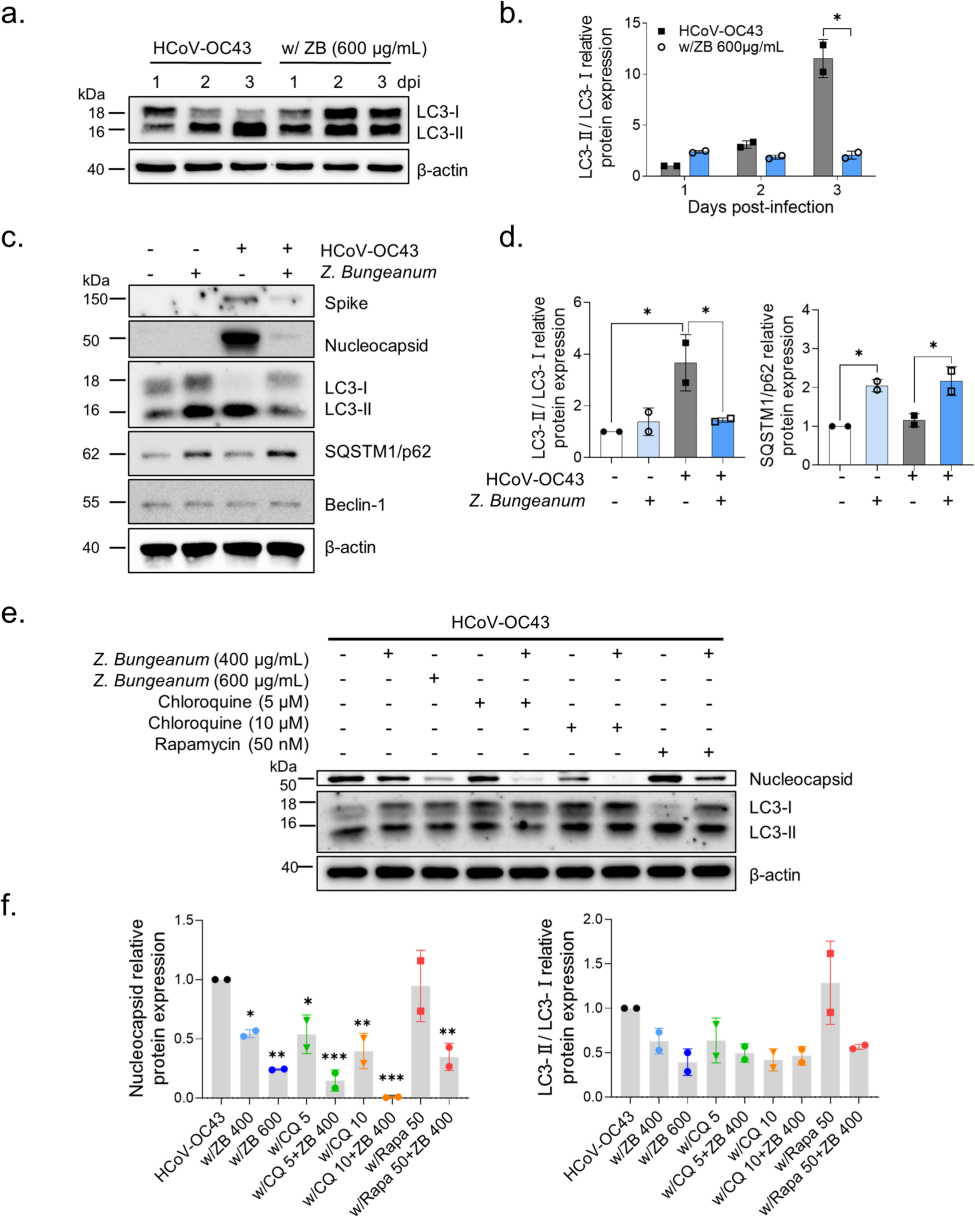
*Z. bungeanum* impeded HCoV-OC43–induced autophagic flux. **a** and **b** LC3 and β-actin were detected in HCoV-OC43–infected MRC-5 cells treated with *Z. bungeanum* (ZB, 600 μg/mL) or vehicle for 24 h by western blotting at 1–3 dpi (**a**). Relative LC3-II/LC3-I ratio (**b**). **c** and **d** Expression of viral proteins (spike and nucleocapsid) and autophagy-related proteins (LC3, SQSTM1/p62, and Beclin-1) was examined in uninfected or virus-infected MRC-5 cells treated with *Z. bungeanum* or vehicle for 24 h at 2 dpi (**c**). The relative LC3-II/LC3-I ratio was presented and SQSTM1/p62 expression were normalized to β-actin (**d**). **e** and **f** Nucleocapsid and LC3 protein expression was measured in HCoV-OC43–infected MRC-5 cells treated with *Z. bungeanum*, chloroquine (CQ), and rapamycin (Rapa) individually or in combination by western blotting at 2 dpi (**e**). The relative expression of nucleocapsid protein was normalized to β-actin and the LC3-II/LC3-I ratio was presented. **f**. Student’s *t*-test or One-way ANOVA with Dunnett’s multiple comparison test was used to assess statistical significance: *p < 0.05, **p < 0.01, ***p < 0.001. Data represent two independent experiments

Prior studies revealed that HCoV-OC43 infection induced autophagic flux in MRC-5 cells, and *Z. bungeanum* modulated the autophagic process [17, 24, 25]. We examined whether *Z. bungeanum* modulates HCoV-OC43–induced autophagy (Fig. 4a, b). LC3-I (18 kDa) is lipidated in its phosphatidylethanolamine-conjugated form. The conversion of LC3-I to LC3-II (16 kDa), resulting in a higher LC3-II/LC3-I ratio, reflects autophagic flux during viral infection [30]. However, *Z. bungeanum* extract treatment prevented the conversion of LC3-I to LC3-II induced by the virus, suggesting that *Z. bungeanum* disturbs virus-induced autophagic flux. We also examined the expression of autophagy-related and viral proteins at 2 dpi (Fig. 4c, d). *Z. bungeanum* decreased the LC3-II/LC3-I ratio and reduced that of the viral spike and nucleocapsid proteins. *Z. bungeanum* induced the accumulation of SQSTM1/p62, indicating that autophagic degradation was inhibited [31]. However, the expression of beclin-1, which initiates autophagy [32], was not changed by *Z. bungeanum* exposure. These data confirmed that *Z. bungeanum* interrupted the HCoV-OC43–induced autophagic flux independently of beclin-1 expression by inhibiting viral protein expression.

To investigate whether the ability of *Z. bungeanum* to impede autophagic flux contributes to the antiviral activity, we used the autophagic inhibitor chloroquine [33] or the autophagic inducer rapamycin during HCoV-OC43 infection (Fig. 4e, f) [34]. Chloroquine concentration-dependently reduced the expression of the nucleocapsid protein and the LC3-II/I ratio. Co-treatment with chloroquine and *Z. bungeanum* (400 µg/mL) led to a strong antiviral effect through their synergistic action and their ability to reduce the LC3-II/LC3-I ratio, suggesting the impairment of autophagic flux. Rapamycin-induced autophagic flux increased the expression of viral proteins. Co-treatment with *Z. bungeanum* and rapamycin reduced the LC3-II/LC3-I ratio and suppressed the expression of viral proteins. These results suggest that the ability of *Z. bungeanum* to impair virus-induced autophagic flux contributes to its antiviral activity.

### Z. bungeanum induces autolysosome accumulation and impairs lysosomal activity

**Fig. 5:**
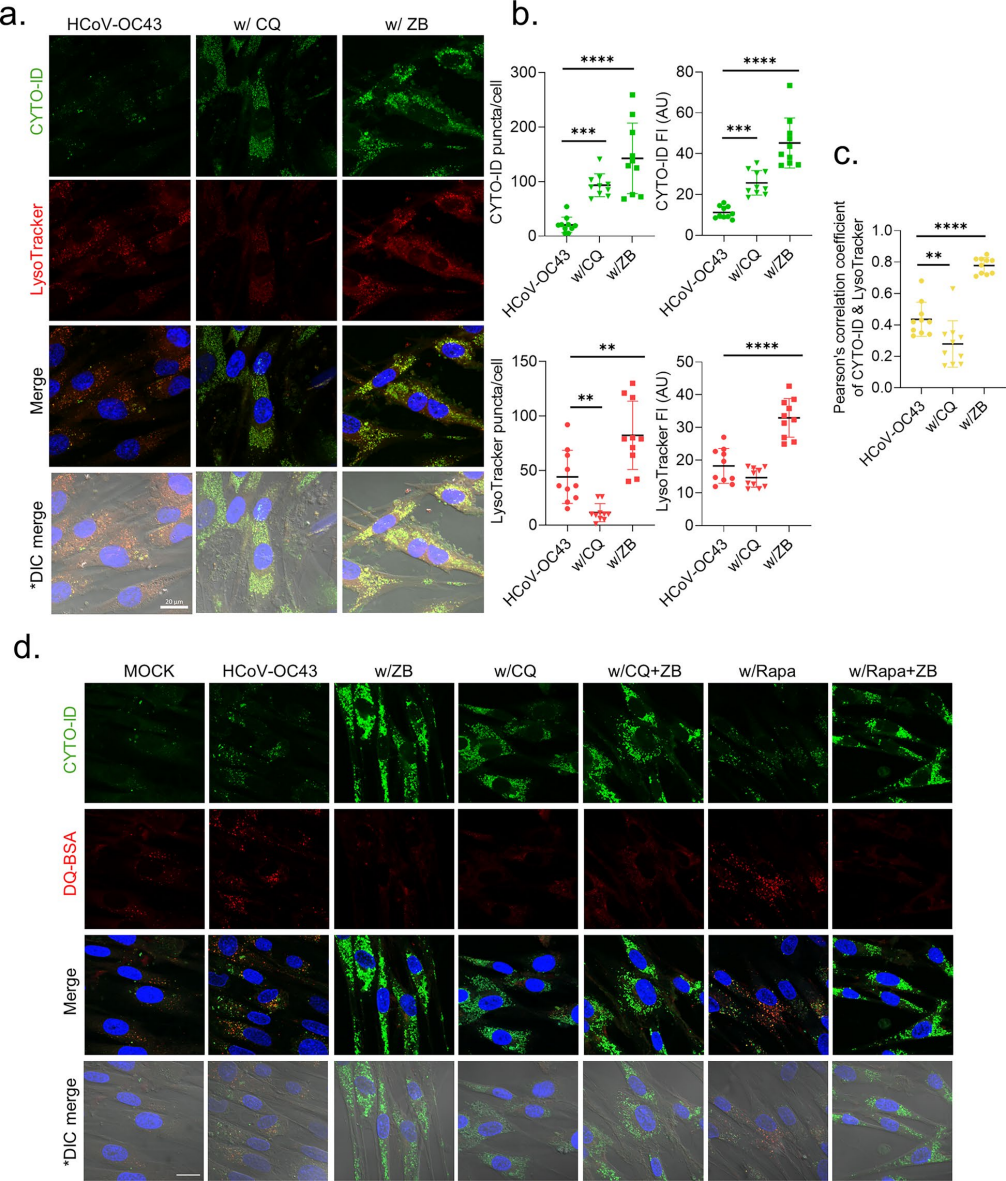
*Z. bungeanum* induced autolysosome accumulation and impaired lysosomal degradation. **a** CYTO-ID (green) and LysoTracker™ Deep Red (red) were used to visualize autophagic vacuoles and lysosomes, respectively, in HCoV-OC43–infected MRC-5 cells treated with 10 μM chloroquine (CQ) or 400 μg/mL *Z. bungeanum* (ZB) at 2 dpi. CYTO-ID, green; LysoTracker, red. **b** The puncta and fluorescence intensity (FI) of CYTO-ID (upper panels) and the puncta and fluorescence intensity (FI) of LysoTracker-stained cells (lower panels) were measured by Fiji/ImageJ. **c** Colocalization was quantified using Pearson’s correlation coefficient between CYTO-ID and Lysotracker puncta by Fiji/ImageJ. Data are presented as arbitrary units (AU). Statistical significance was assessed using one-way ANOVA with Dunnett’s multiple comparison test: **p < 0.01, ***p < 0.001, ****p < 0.0001 versus HCoV-OC43 group. **d** Activity of lysosomal protease was determined by DQ-BSA staining (red) with CYTO-ID staining (green) of autophagic vacuoles in uninfected MRC-5 cells (MOCK) or MRC-5 cells infected with HCoV-OC43 in the presence of ZB (400 μg/mL), CQ (10 μM), and Rapa (50 nM) individually or in combination at 2 dpi. Hoechst 33342, blue; differential interference contrast (DIC), merged image. Scale bar: 20 μm

To further examine the impairment of autophagic flux by *Z. bungeanum*, we stained autophagic vacuoles (pre-autophagosomes, autophagosomes, and autolysosomes) with the CYTO-ID and acidic lysosomes with LysoTracker Deep Red (Fig. 5a, b). Autophagosomes fuse with lysosomes to form autolysosomes [31], and chloroquine was reported to neutralize the lysosomal pH and inhibit autophagosome–lysosome fusion [33]. Both *Z. bungeanum* and chloroquine induced autophagic vacuole accumulation, as indicated by the increased number of puncta and the increased fluorescence intensity compared with the findings in virus-infected cells at 2 dpi. Chloroquine reduced the number of LysoTracker puncta through its neutralization of lysosome pH, whereas *Z. bungeanum* increased the number of LysoTracker puncta and the fluorescence intensity compared with the findings in the virus-infected cells. Moreover, *Z. bungeanum* induced the colocalization of CYTO-ID and LysoTracker puncta, implying the formation of autolysosomes (Fig. 5c). These data indicated that *Z. bungeanum* induced autolysosome accumulation.

Autophagosomes are degraded after the formation of autolysosomes during autophagy process. We determined the capacity of *Z. bungeanum* to inhibit lysosomal degradation in autophagic vacuoles using CYTO-ID and DQ-BSA, a substrate that exhibits fluorescence when degraded by lysosomal proteases [35] (Fig. 5d). HCoV-OC43 infection increased the numbers of overlapping CYTO-ID and DQ-BSA puncta compared with the findings in uninfected cells, suggesting virus-induced autophagic flux during the entire process up to lysosomal degradation. *Z. bungeanum* decreased the number of DQ-BSA puncta in CYTO-ID–stained autophagic vacuoles, suggesting the impairment of virus-induced lysosomal degradation. Rapamycin-induced lysosomal degradation as demonstrated by DQ-BSA staining, but this staining disappeared upon *Z. bungeanum* treatment. These findings suggest that *Z. bungeanum* impaired lysosomal degradation in the accumulated autophagic vacuoles, thereby interfering with HCoV-OC43–induced autophagic flux.

### Hydroxy-α-sanshool and p-coumaric are the active antiviral compounds of Z. bungeanum

**Fig. 6:**
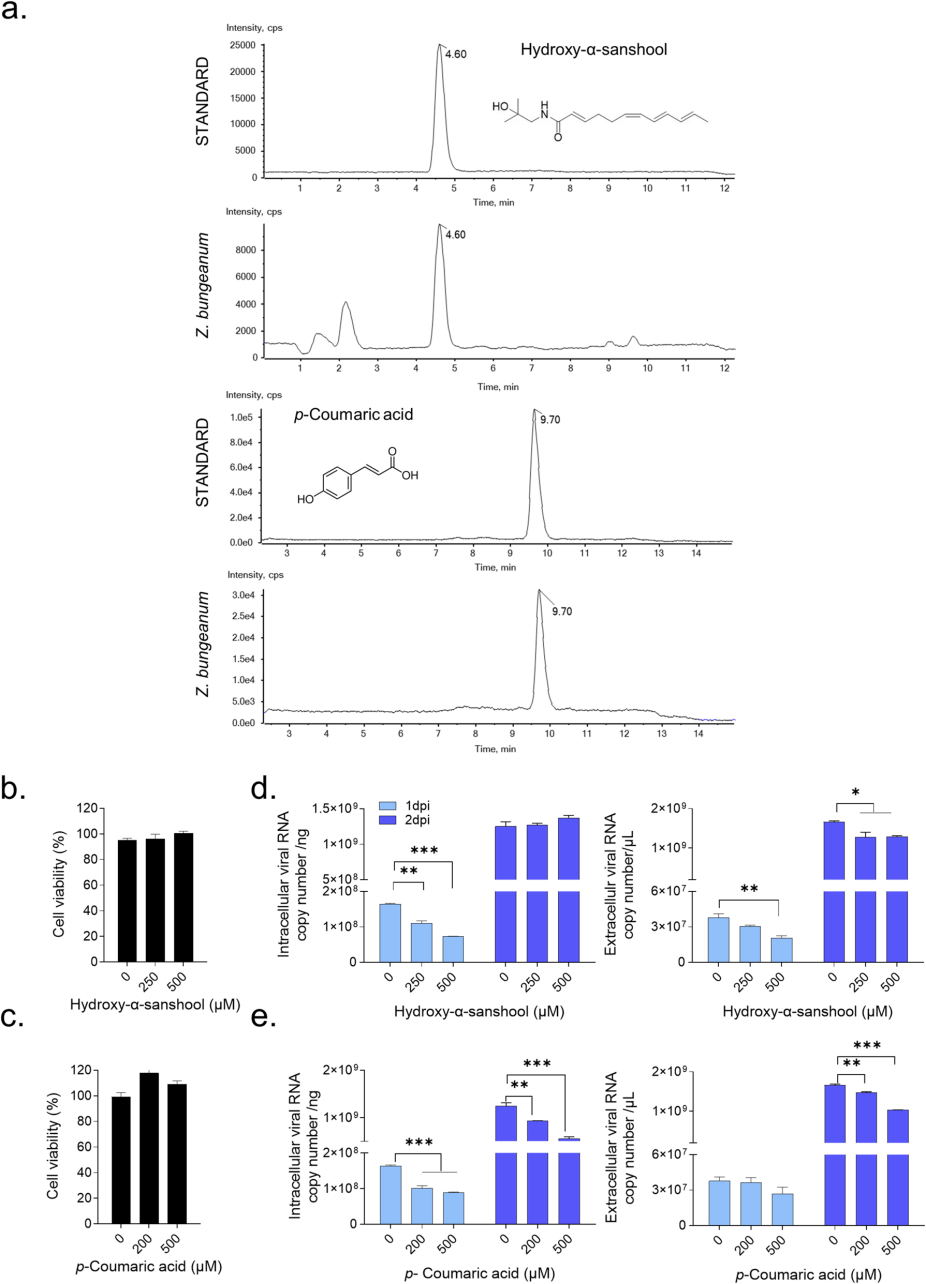
Hydroxy-α-sanshool and *p*-coumaric acid are the active anticoronavirus compounds of *Z. bungeanum*. **a** Representative total ion chromatogram of hydroxy-α-sanshool and *p*-coumaric acid as standard compound and in the 30% ethanol extract of *Z. bungeanum*. **b** and **c** MRC-5 cells were treated with hydroxy-α-sanshool (**b**) and *p*-coumaric acid (c) for 2 days, and cell viability was measured by the MTS assay. **d** and **e** Viral RNA copy numbers were analyzed by qRT-PCR in HCoV-OC43–infected cell lysate (left panel) and supernatant (right panel) treated with hydroxy-α-sanshool (**d**) and *p*-coumaric acid (**e**). One-way ANOVA with Dunnett’s multiple comparisons test was used for statistical analysis: *p < 0.05, **p < 0.01, ***p < 0.001 versus 0 group. Data represent two independent experiments

Using UHPLC-QTOF MS, we confirmed hydroxy-α-sanshool and *p*-coumaric acid (trans-4-hydroxycinnamic acid) as components in the 30% ethanol extract of *Z. bungeanum* (Fig. 6a). After we determined that hydroxy-α-sanshool and *p*-coumaric acid did not induce cytotoxicity at concentrations up to 500 µM (Fig. 6b, c), the antiviral activity of these compounds was examined in HCoV-OC43–infected MRC-5 cells. Hydroxy-α-sanshool effectively reduced the number of intracellular and extracellular viral RNA copy at 1 dpi. Likewise, *p*-coumaric acid reduced the number of intracellular and extracellular viral RNA copy at 2 dpi (Fig. 6d, e). We suggested that hydroxy-α-sanshool and *p*-coumaric were the active antiviral compounds of *Z. bungeanum* against HCoV-OC43.

## Discussion

*Z. bungeanum* has been traditionally used in herbal medicine owing to its pharmacological activities such as anti-inflammatory and antitumor effects, in addition to its uses as a spice because of its unique flavor [20, 21, 36]. In this study, we discovered the anticoronaviral activity of *Z. bungeanum* by a CPE reduction activity screening of natural product extracts and elucidated its mechanism of action as well as the active antiviral components. *Z. bungeanum* targets the early phase of the virus life cycle by inhibiting viral entry and impairs virus-induced autophagy flux by inducing the accumulation of impaired autolysosomes.

Viruses have been reported to utilize autophagy for their replication and immune evasion [11–13]. HCoV-OC43 infection induces autophagic flux, whereas the impairment of flux effectively reduces viral replication [17]. We demonstrated that HCoV-OC43 infection induced the conversion of LC3-I to LC3-II and the accumulation of autophagic vacuoles together with lysosomal degradation, reflecting autophagic flux. However, *Z. bungeanum* interrupted virus-induced autophagy by inhibiting the conversion of LC3-I to LC3-II and increasing the expression of SQSTM1/p62, in addition to inducing autophagic vacuole accumulation, consistent with the effects of the autophagy inhibitor chloroquine. The synergistic antiviral effects of the combination of chloroquine and *Z. bungeanum* highlighted that blocking autophagic flux inhibits HCoV-OC43 replication. Conversely, the autophagy inducer rapamycin increased the expression of viral proteins.

Beclin-1 plays a critical role in initiating autophagy and stimulating autolysosome generation [31, 32]. HCoV-NL63 membrane-associated papain-like protease PLP2 (PLP2-TM) interacted with beclin-1 to induce autophagy and negatively regulate the interferon response, and MERS-CoV infection induced beclin-1 ubiquitination and degradation [37, 38]. However, neither HCoV-OC43 nor *Z. bungeanum* altered beclin-1 expression under our conditions, suggestion the existence of noncanonical beclin-1–independent autophagy similar to that in poliovirus [39]. Chloroquine modulates autophagic flux by altering lysosome acidification and inhibits autophagosome–lysosome fusion [33], consistent with our findings of increased CYTO-ID staining in autophagosomes without LysoTracker co-staining. However, *Z. bungeanum* induced the accumulation of impaired autolysosomes in HCoV-OC43–infected MRC-5 cells as demonstrated by CYTO-ID and LysoTracker co-staining in autophagic vacuoles lacking lysosomal activity, as demonstrated by the lack of DQ-BSA staining. These data suggested that *Z. bungeanum* impaired virus-induced autophagic flux after autophagosome–lysosome fusion via different mechanisms from chloroquine, resulting in the accumulation of autolysosomes with no lysosomal activity. The natural compound, tubeimoside I from *Bolbostemma paniculatum* Franquet was reported to exert antitumor activity as a modulator of autophagic flux through the accumulation of impaired autolysosomes, similar to the effects of *Z. bungeanum* [40].

*Z. bungeanum* is known for its anti-inflammatory and anti-cancer activity, and our study revealed its antiviral activity against HCoV-OC43 [21–23]. We identified the twenty compounds in the 30% ethanol extract of *Z. bungeanum* using UHPLC-QTOF MS/MS, and conducted a screening to find its novel anticoronaviral compounds. Quercetin was reported to have significant antiviral efficacy against HCoV-OC43 [41]. Chlorogenic acid has previously been reported to be effective antiviral activity against SARS-CoV-2 [42]. We also confirmed the antiviral activity of quercetin, however quercetin 3-D-galactoside, chlorogenic acid, and its isomers, 3,5-dicaffeoylquinic acid, neochlorogenic acid, and cryptochlorogenic acid, did not show the antiviral activity against HCoV-OC43 in our experiment (Additional file 1: Figure S1). Hydroxy-α-sanshool and *p*-coumaric acid were identified as the active antiviral components of *Z. bungeanum*. A main alkylamide, hydroxy-α-sanshool is a responsible for the pungency of *Z. bungeanum* [26]. It has diverse biological activities, including antioxidant and hypolipidemic activity, but no studies have described its antiviral effects [43]. *p*-Coumaric acid exhibited anti-rhinoviruses activity by suppressing viral entry into cells, in addition to anti-cancer, anti-inflammatory, and antioxidative activities [44, 45]. We found that these compounds significantly inhibited HCoV-OC43 infection by reducing viral RNA copy numbers, however these compounds did not impair virus-induced autophagic flux (Additional file 1: Figure S2). Further study is needed to clarify the mechanism of these compounds and the active components in *Z. bungeanum* that modulate autophagic flux. Moreover, autophagy is a complex, multi-layered process that may involve various signaling pathways, and further studies are needed to clarify the detailed molecular mechanisms underlying the relationship between the autophagy impairment and the antiviral effects of *Z. bungeanum*.

## Conclusion

In conclusion, *Z. bungeanum* exhibits remarkable anticoronaviral effects by inhibiting viral entry and interrupting virus-induced autophagic flux by inducing the accumulation of autolysosomes, supporting its potential as a potent antiviral agent. Hydroxy-α-sanshool and *p*-coumaric acid were identified as the active antiviral components of *Z. bungeanum*. Further studies are needed to verify the in vivo efficacy of *Z. bungeanum*, pre-clinical, and clinical trials to facilitate the development of anticoronaviral therapeutics.

## Supplementary Information

Additional file 1

## Data Availability

The data used to support the findings of this study are included within the article.

## Abbreviations

ATCC: American type culture collection
CPE: Cytopathic effect
DMSO: Dimethyl sulfoxide
dpi: Days post-infection
HCoV: Human coronavirus
MEM: Minimum essential medium
UHPLC-QTOF MS: Ultra-performance liquid chromatography quadrupole time-of-flight mass spectrometry
