# Supplementary material for: Sichuan pepper, Zanthoxylum bungeanum Maxim., suppresses human coronavirus OC43 infection by inhibiting viral entry and impairing autolysosome accumulation
Source: Chin Med. 2026 Apr 2;21:108. doi: 10.1186/s13020-026-01388-y (PMC13045090; doi:10.1186/s13020-026-01388-y)

**Supplementary Methods**

**UHPLC-QTOF MS analysis to identify chemical components in the 30% ethanol extract of *Z. bungeanum***

Chromatographic analysis was performed to identify representative components in the 30% ethanol extract of *Z. bungeanum*. Chromatographic separation and mass spectrometry were performed using a Thermo Scientific Vanquish UHPLC system (Thermo Fisher Scientific) with an Acquity UPLC HSS T3 column (2.1 mm × 100 mm, 2.7 μm; Waters) coupled to a Triple TOF5600^+^ mass spectrometer system (QTOF MS/MS, SCIEX) using information-dependent acquisition (IDA) scan. The parameters were as follows: mass range, 50–1800 m/z; ion spray voltage, 4.5 kV; source temperature, 550°C for positive ion mode and 450°C for negative ion mode; declustering potential, 30 V; nitrogen as the nebulizer gas, 50 L/min; heater gas, 50 L/min; curtain gas, 25 L/min; and collision energy, 10 eV. The gradient conditions for chromatographic separation, including 0.1% formic acid in water as eluent A and 0.1% formic acid in acetonitrile as eluent B, were as follows: 0–2 min, 5% B; 2–5 min, 5%–15% B; 5–12 min, 15%–35% B; 12–18 min, 35%–50% B; 18–20 min, 50%–100% B; 20–25 min, 100% B; followed by equilibration with 5% B for 4 min at a 0.4-mL/min flow rate. Column temperature was maintained 40°C, and the autosampler was kept at 4°C. Injection volume was 2 μL. Molecular formulas were generated using PeakView 2.2 and MasterView software (SCIEX). Expected compounds were identified by comparing chromatographic peaks to chemical constituents reported in the literature (https://nifds.go.kr/nhmi/srcbk/crshm/list.do), reference standards, in-house MS/MS libraries, and/or online databases, such as GNPS, MASS bank, and Metlin.

**Development and validation of** **a reliable UHPLC-QTOF MS/MS method for simul-taneous quantification in the 30% ethanol extract of *Z. bungeanum***

The ultra-high-performance liquid chromatography (UHPLC) system (Vanquish, Thermo Fisher Scientific, Sunnyvale, CA, USA) was performed to separate hydroxy-α-sanshool and p-Coumaric acid at 40℃ using Waters ACCQUITY UPLC CSH C18 column (2.1 x 100mm, 1.7µm). A gradient elution for chromatographic separation used 0.1% formic acid in water as eluent A and 0.1% formic acid in acetonitrile as eluent B was as follows: 0-1 min; 15% B, 1-5 min; 15-25% B, 5-6 min; 25-45% B, 6-10 min; 45-50% B, 10-11 min; 50-100% B, 11-15 min; 100% B and equilibration with 5% B for 4.5 min at a flow rate of 0.3 mL/min. The auto-sampler was maintained at 4℃. The injection volume of each sample solution was 2 μL. MS/MS analysis was performed on a TripleTOF5600^+^ Mass Spectrometer (Sciex, Foster City, CA, USA) equipped with A Turbo-V IonSpray. An Electrospray Ionization (ESI) source in the positive and negative ion mode was utilized for identification of two components and quantitation using IDA scan and MRM^HR^ scan concurrently. The parameters were as follows: mass range 50–1200 m/z, ion spray voltage, 4.5 kV; source temperature, 450 °C; declustering potential, 50 V; nitrogen as nebulizer gas, 50 L/min; heater gas, 50 L/min; curtain gas, 25 L/min; and collision energy, 10 eV. The MS and MS/MS Data acquisition and processing for qualitative analysis were carried out using Analyst TF 1.7, PeakVeiw 2.2 and MasterView (SCIEX, Foster City, CA, USA). The MS/MS data for qualitative analysis were processed using PeakView and MasterView software to identify and confirm components in the 30% ethanol extract the retention time, accurate m/z value, isotope distribution, and fragment ions comparing reference standards. For quantitative analysis, we used MultiQuant software. The amounts of each marker compound were quantified using the corresponding calibration curves of the reference standards.

**Supplementary Results**

The developed UHPLC-QTOF MRM (MRM^HR^) systems were utilized for quantitative analysis of marker compounds. The detection of two analytes and IS by MRM^HR^ mode was highly selective with no interference. The representative total ion chromatograms (TIC) in MRM^HR^ analysis of reference standards and samples are shown in **Fig. 6a.** Further details on the mass spectrometry parameters and conditions for MRM^HR^ scan can be found in **Table S1**. The present study set out to validate a number of key parameters of the UHPLC-QTOF MRM method, including specificity, limits of detection (LODs) and limits of quantitation (LOQs), system suitability, linearity, accuracy and precision. The validation process was conducted in accordance with European Medicines Agency (EMA) guidelines and the relevant guidelines [J. Pharm Res. (2013) 6, 53-60 and J. Pharmaceutical and Biomedical Analysis (2017) 139, 165-178.].

The linearity of the calibration curve was determined by plotting the peak area ratio (y) of analyte to IS versus the nominal concentration (x) of analyte. The calibration curve test range was found to be adequate for the concentrations observed from the analysis of samples, and two analytes showed linearity over the low and high concentration range with r2 > 0.999. The linear ranges, regression equations and correlation coefficients are shown in **Table S2**. The LOQs of analytes were 0.42 μg/mL for hydroxy-α-sanshool and 3.84 ng/mL for *p*-coumaric acid. To ensure robust and reliable MRM-MS method, system suitability testing (SST) in MRM was evaluated using RT, peak area and width, and peak drift and variation. These metrics were evaluated using a standard at 25 μg/mL for hydroxy-α-sanshool and 250 ng/mL for *p*-coumaric acid. The intra-day and inter-day precision and accuracy results for the tested samples were all within the acceptable criteria (RSD% <15%; RE% <±15%), as shown in **Table S3**. This indicates that the present method is highly reproducible and accurate. TOF-MRM mode could provide sensitivity and large linear dynamic range often needed for quantitative studies. This

analytical method was then applied to quantitate contents of markers from the 30% ethanol extract. As shown in **Table 2**, the content of hydroxy-α-sanshool was found to be 144.13 ± 2.15 mg/g, and the content of *p*-coumaric acid was found to be 0.09 ± 0.00 mg/g.

**Table S1.** MS parameters and conditions in MRM mode for two chemical markers and internal standard (IS)

| **Compounds** | **Q1 Mass** | **Q3 Mass** | **Time** | **DP** | **CE** | **Ionization mode** |
| --- | --- | --- | --- | --- | --- | --- |
|  | **(Da)** | **(Da)** | **(msec)** | **(volts)** | **(volts)** |  |
| Hydroxy-α-Sanshool (HαS) | 264.1 | 246.1 | 300 | 31 | 13 | positive mode |
| IS_Butylparaben | 195.1 | 139.0 | 300 | 31 | 13 | positive mode |
| *p*-Coumaric acid (*p*-CA) | 163.0 | 119.0 | 300 | -30 | -20 | negative mode |
| IS_Butylparaben | 193.1 | 92.0 | 300 | -30 | -20 | negative mode |

**Table S2**. Method validation parameters for quantification of the 30% ethanol extract

| Method validation  parameters | Results | |
| --- | --- | --- |
|  | Hydroxy-α-sanshool | *p*-Coumaric acid |
| **LOD** | 0.13 (μg/mL) | 1.15 (ng/mL) |
| **LOQ** | 0.42 (μg/mL) | 3.84 (ng/mL) |
| **Linear range** | 1 ~ 100 (μg/mL) | 10 ~ 1000 (ng/mL) |
| **System suitability (% CV, n=6)** | 0.44 | 0.69 |
| **Calibration curve** | 0.09131x + 0.11239 | 0.00576x + 0.02150 |
| **R^2^** | 0.99945 | 0.99935 |
| **Retention time (min)** | 9.70 | 4.60 |

**Table S3.** Intra-day (within-run) and Inter-day (between-run) precision and accuracy % of measured concentrations of analyte spiked in the 30% ethanol extract

| Analytes | Nominal  Concentration | Intra-day (within- run) | | | Inter day (Between-run) | | |  |
| --- | --- | --- | --- | --- | --- | --- | --- | --- |
|  |  | Measured Concentration (Mea ± SD), (µg/mL) | Precision CV  (%) | Accuracy  (%) | Measured Concentration (Mea ± SD), (ng/mL) | Precision CV (%) | Accuracy (%) |  |
|  |  |  |  |  |  |  |  |  |
| Hydroxy-α-sanshool | LQC | 30.50 ± 0.19 | 0.61 | 98.32 | 30.91 ± 0.43 | 1.39 | 99.01 |  |
|  | MQC | 51.96 ± 0.63 | 1.20 | 101.84 | 51.70 ± 0.41 | 0.79 | 100.95 |  |
|  | HQC | 78.07 ± 0.19 | 0.25 | 102.69 | 77.00 ± 1.00 | 1.30 | 101.03 |  |
| *p*-Coumaric acid | LQC | 242.10 ± 2.46 | 1.02 | 97.59 | 243.70 ± 2.46 | 2.46 | 98.02 |  |
|  | MQC | 416.33 ± 6.12 | 1.47 | 100.06 | 420.82 ± 4.07 | 4.07 | 101.01 |  |
|  | HQC | 620.17 ± 3.88 | 0.63 | 99.06 | 620.93 ± 1.75 | 1.75 | 99.09 |  |

* Intra-day values expressed as mean ±SD is the result of single day triplicate injection (n =3 injections); The inter-day values expressed as mean ±SD is the result of three-day triplicate injection per analyte in LC-MS/MS (n =3 replicates ×3 days =9 injections per analyte).

(LQC- Low quality control; MQC- Mid quality control; HQC- High quality control.)

**Supplementary Figures**

**Figure S1.** Screening for the antiviral activity *of Z. bungeanum* components against HCoV-OC43

**
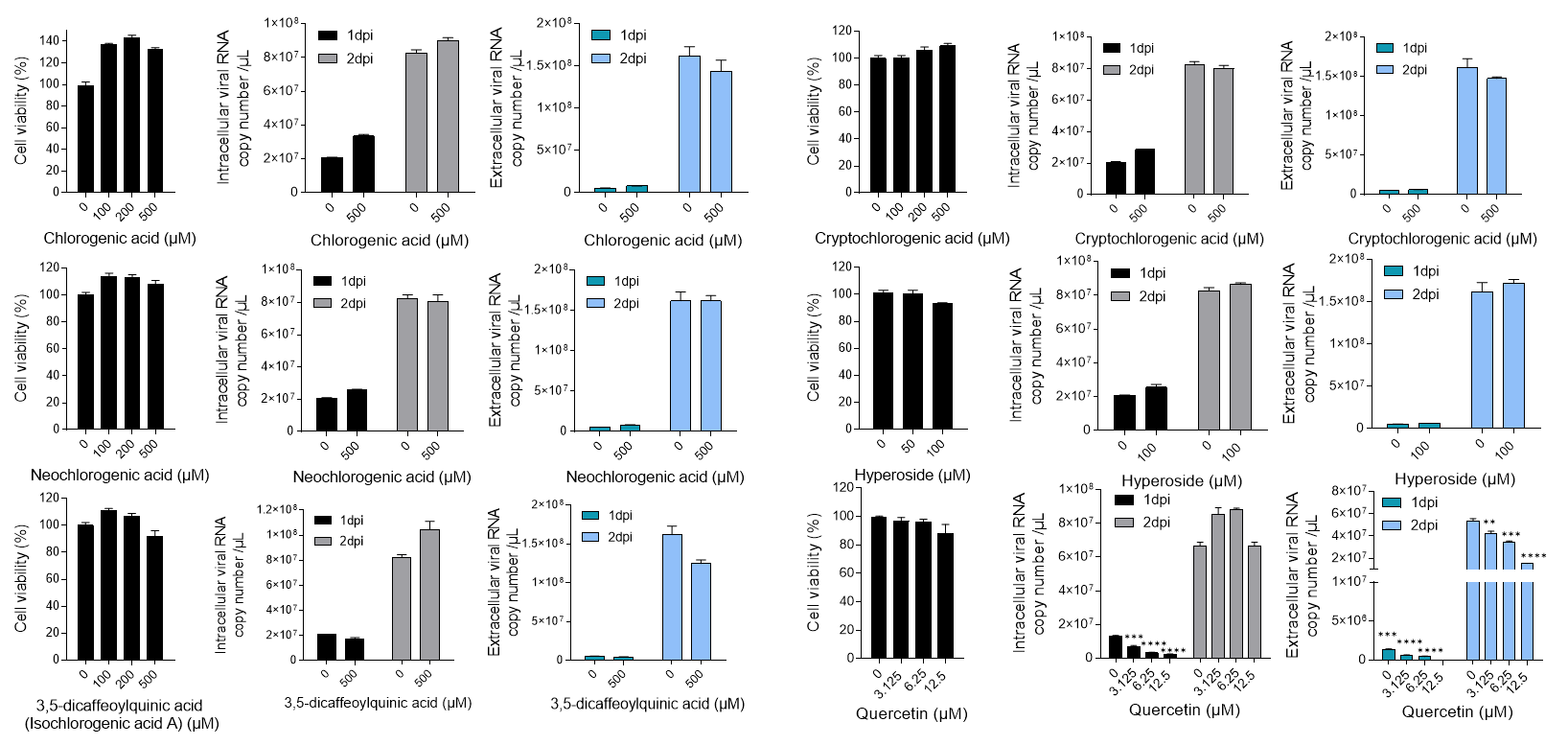
**

**Figure S2.** The expression levels of LC3 and SQSTM1/p62 in HCoV-OC43 infected MRC5 cells treated with p-coumaric acid (*p*CA) and hydroxy-α-sanshool (HαS) for 2 days

**
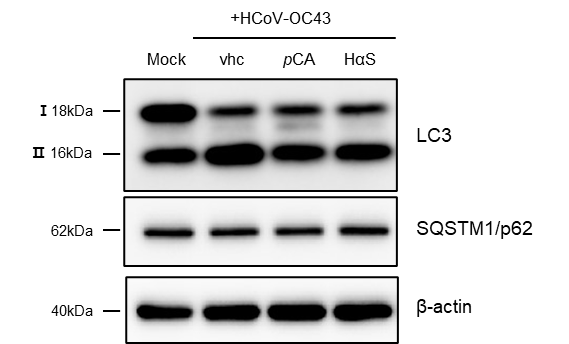
**

**Figure S3.** Whole uncropped images of the original western blots in Figure 2C


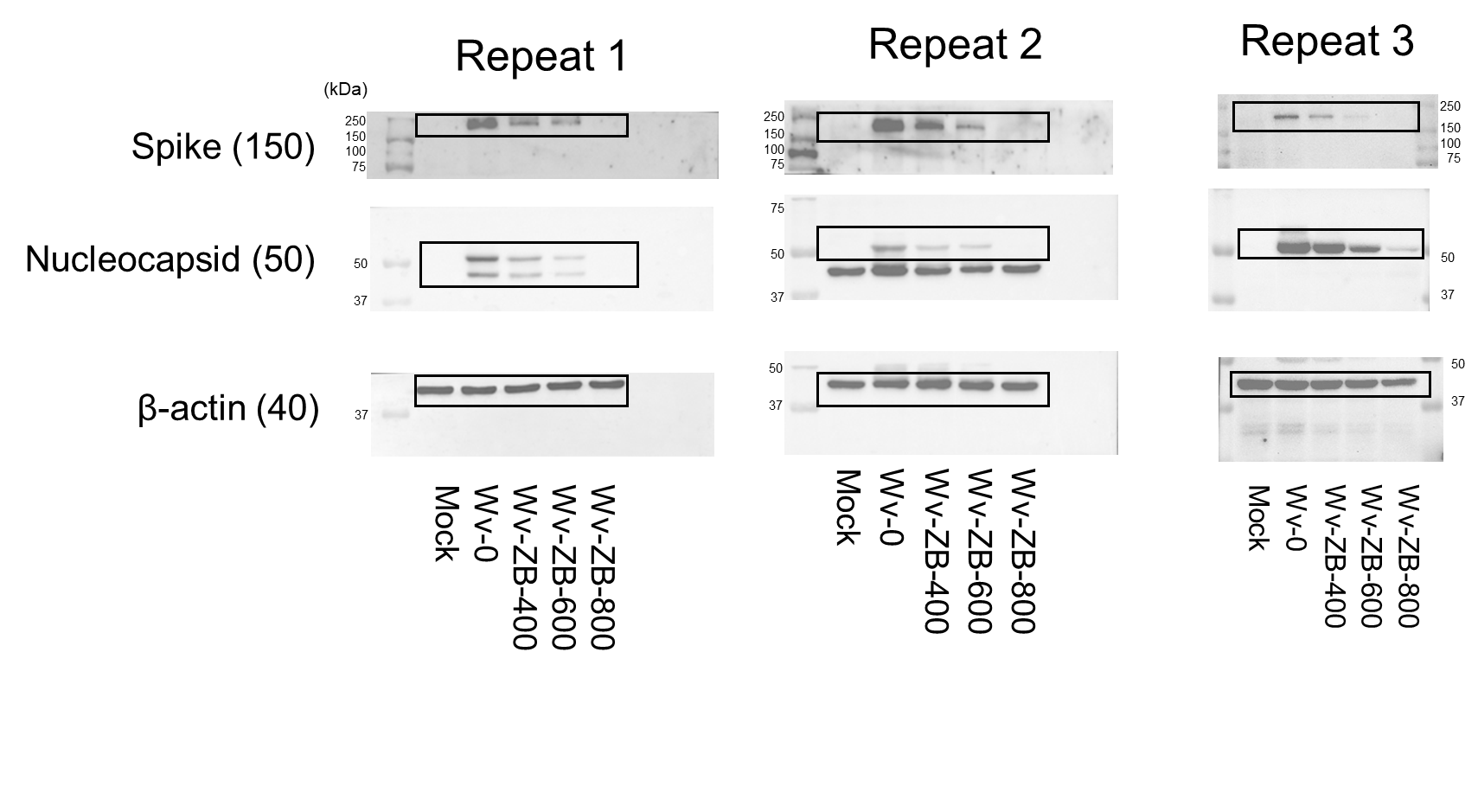


**Figure S4.** Whole uncropped images of the original western blots in Figure 4A


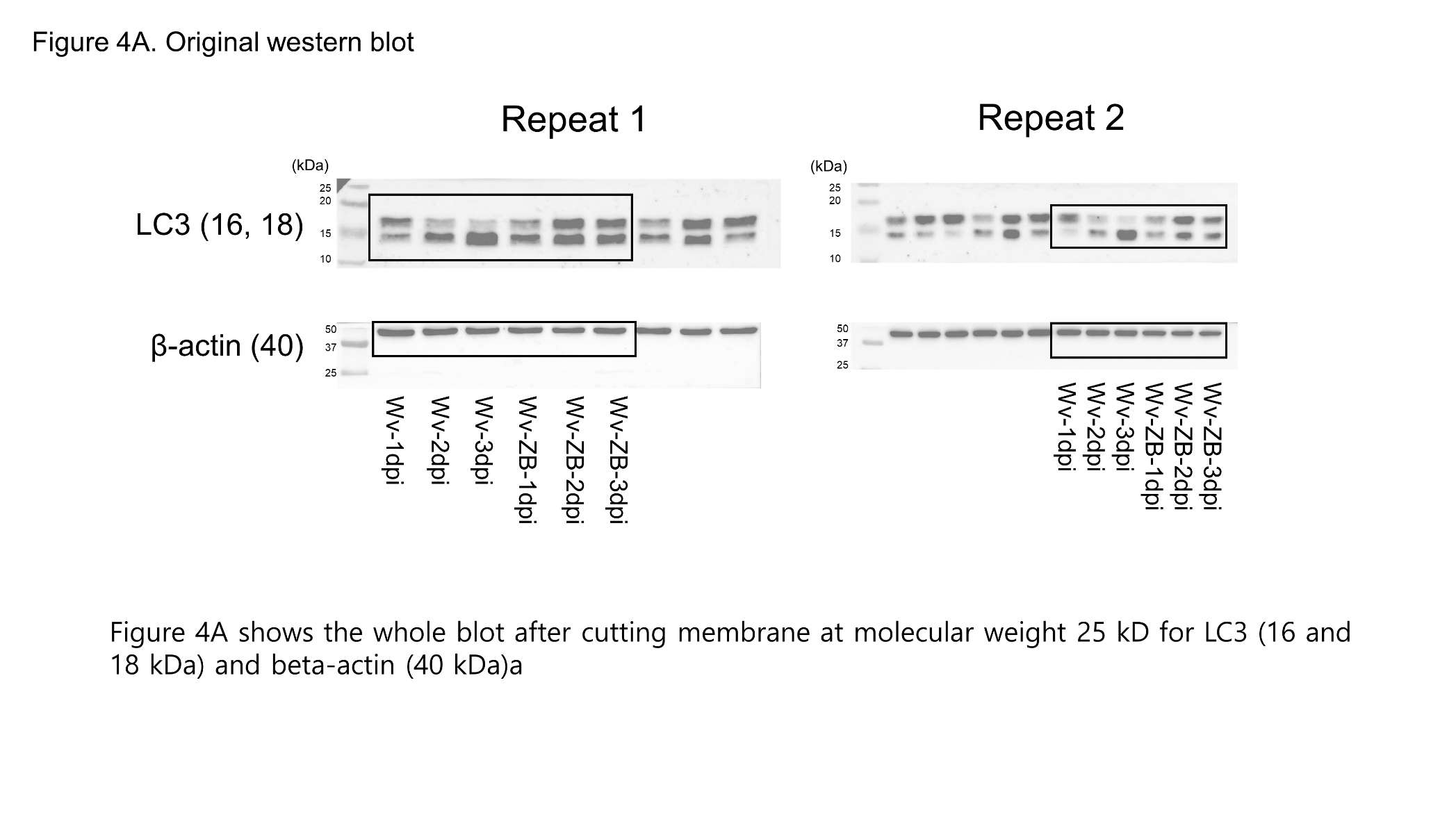


**Figure S5.** Whole uncropped images of the original western blots in Figure 4C


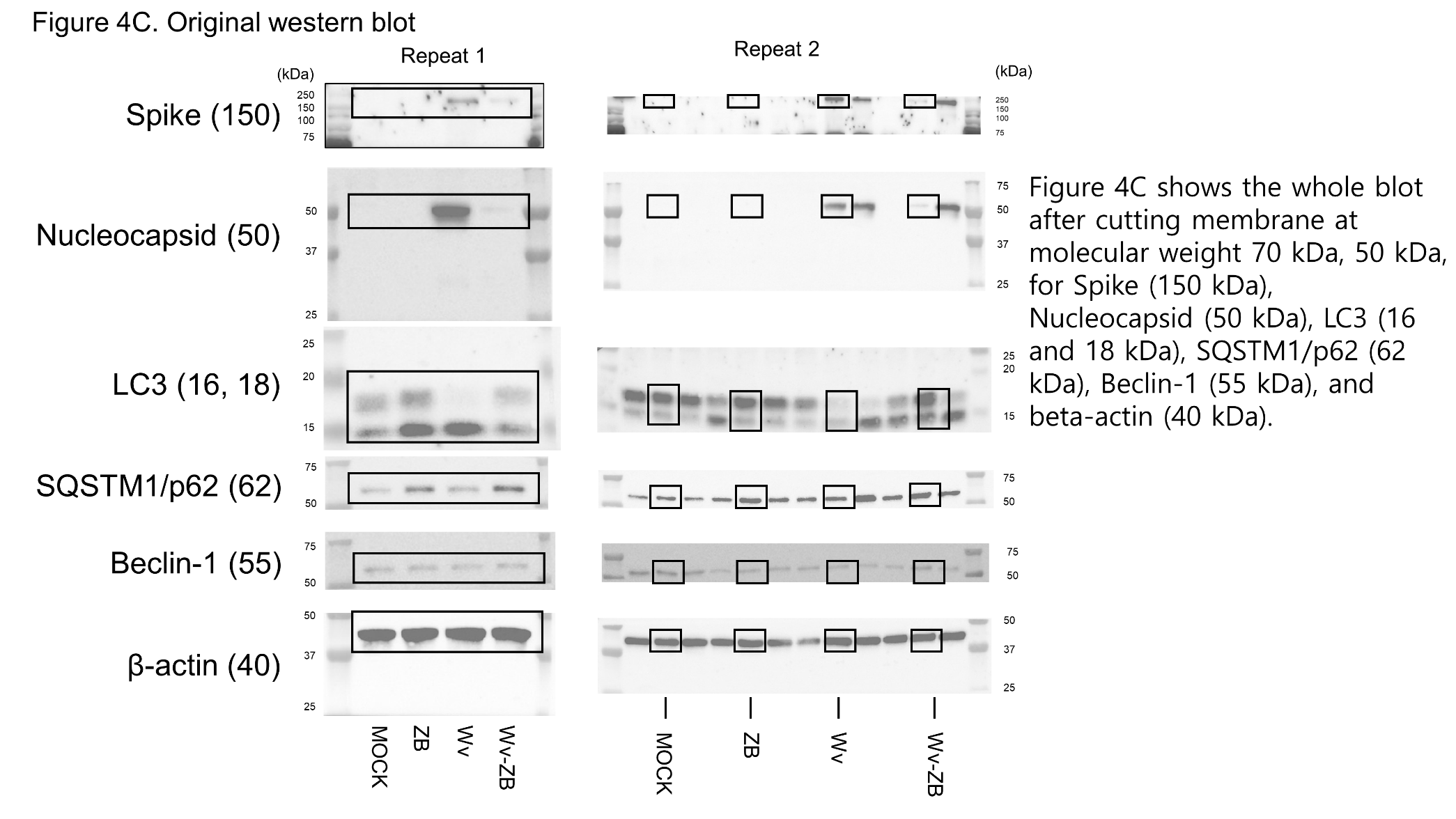


**Figure S6.** Whole uncropped images of the original western blots in Figure 4E


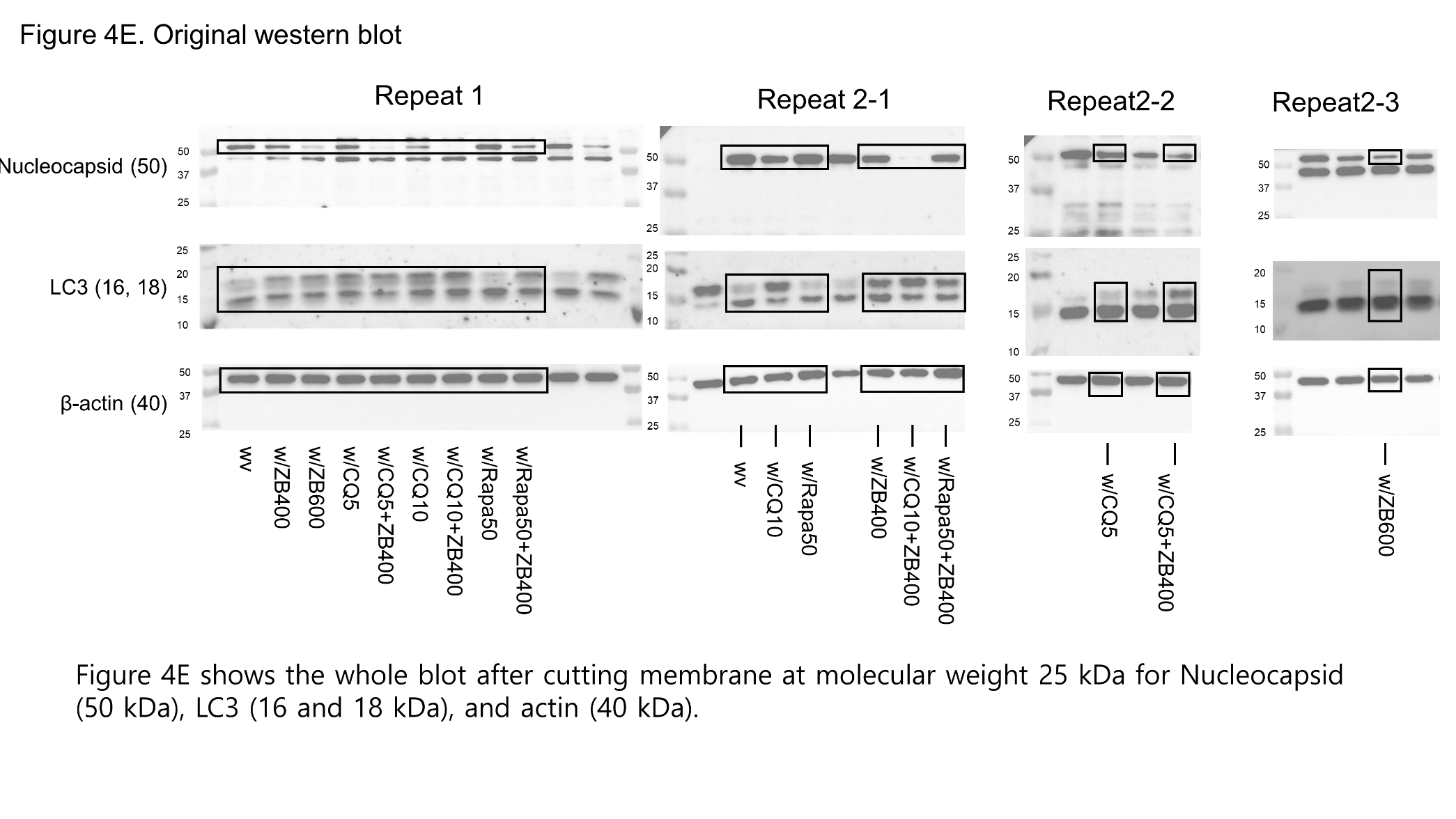

Supplement: Supplementary file 1 — Additional file 1 [file 13020_2026_1388_MOESM1_ESM.docx]
